# Clinical Implications of IL-32, IL-34 and IL-37 in Atherosclerosis: Speculative Role in Cardiovascular Manifestations of COVID-19

**DOI:** 10.3389/fcvm.2021.630767

**Published:** 2021-08-06

**Authors:** Ching Chee Law, Rajesh Puranik, Jingchun Fan, Jian Fei, Brett D. Hambly, Shisan Bao

**Affiliations:** ^1^School of Biomedical Engineering, The University of Sydney, Sydney, NSW, Australia; ^2^Department of Cardiology, Royal Prince Alfred Hospital, Sydney, NSW, Australia; ^3^School of Public Health, Gansu University of Chinese Medicine, Lanzhou, China; ^4^Shanghai Engineering Research Centre for Model Organisms, SMOC, Shanghai, China

**Keywords:** IL-32, IL-34, IL-37, implication, COVID-19

## Abstract

Atherosclerosis, which is a primary cause of cardiovascular disease (CVD) deaths around the world, is a chronic inflammatory disease that is characterised by the accumulation of lipid plaques in the arterial wall, triggering inflammation that is regulated by cytokines/chemokines that mediate innate and adaptive immunity. This review focuses on IL-32, -34 and -37 in the stable vs. unstable plaques from atherosclerotic patients. Dysregulation of the novel cytokines IL-32, -34 and -37 has been discovered in atherosclerotic plaques. IL-32 and -34 are pro-atherogenic and associated with an unstable plaque phenotype; whereas IL-37 is anti-atherogenic and maintains plaque stability. It is speculated that these cytokines may contribute to the explanation for the increased occurrence of atherosclerotic plaque rupture seen in patients with COVID-19 infection. Understanding the roles of these cytokines in atherogenesis may provide future therapeutic perspectives, both in the management of unstable plaque and acute coronary syndrome, and may contribute to our understanding of the COVID-19 cytokine storm.

## Atherosclerosis

Cardiovascular disease (CVD) is the leading cause of death in the world (1). Cerebrovascular disease and coronary artery disease (CAD) are the most prevalent subtypes of cardiovascular disease that result in a high morbidity as well as large economic burden in developing countries (1). Atherogenesis, referring to the development of atherosclerotic plaques, progresses through endothelial dysfunction; leukocytes recruitment; differentiation of monocytes; formation of foam cells; and proliferation of vascular smooth muscle cells (VSMC) (2). The abnormal steps of atherogenesis are regulated by both innate and adaptive immunity *via* cytokines/chemokines modulating the cross-talk between inflammatory and vascular cells (2, 3). Despite the aggressive management of modifiable risks factors for atherosclerosis, for example, lipid-lowering treatments and anti-hypertensives, which promise effective management for atherosclerosis, the mortality and morbidity of CVD are still rather unacceptably high (4). The *Canakinumab Anti-Inflammatory Thrombosis Outcomes Study* is a large-scaled clinical trial which demonstrates a decrease in major adverse cardiovascular events following anti-IL-1β, antibody treatment, supporting the critical role of inflammation during atherogenesis (5).

## Atherogenesis

Circulating low-density lipoproteins (LDL) are deposited in the intima at lesion-prone sites and undergo oxidative modification to generate oxidised LDL (OxLDL), which is a potent inflammatory mediator that triggers endothelial dysfunction (6, 7). Endothelial cells respond to OxLDL by expressing adhesion molecules such as ICAM-1 and chemokines including monocyte chemotactic protein-1 (MCP-1/CCL2) for recruitment of leukocytes (7, 8). Macrophages perform a protective role to metabolise lipids *via* scavenger receptors that internalise OxLDL and ATP-binding cassette (ABC) transporters A-1 and G-1 that mediate the efflux of OxLDL (9). However, imbalance of cholesterol influx and efflux results in the accumulation of lipids within macrophages, which contributes to foam cells formation (3, 9). Continuous low grade inflammation within the vessel wall subsequently progressively transforms a fatty streak into a fibro-fatty plaque, which is characterised by a fibrous cap covered by a necrotic core within the grossly thickened arterial intima (3, 10). The fibrous cap is formed by proliferating VSMC that migrate from the media, synthesising and releasing extracellular matrix to stabilise the plaque; whereas the necrotic core is formed by apoptotic macrophages/foam cells that have become exhausted by excessive lipid metabolism (3). Thinning of the fibrous cap is induced by inflammatory mediators triggering apoptosis of VSMC and the production of collagenolytic enzymes that degrade the collagen within the cap (11). Ineffective clearance of apoptotic cells contributes to secondary necrosis, releasing damage-associated molecular patterns (DAMP) to sustain the inflammation, thus enlarging the necrotic core (11). These features characterise the unstable symptomatic plaque that is susceptible to rupture, which results in the release of pro-thrombotic materials to cause intra-vascular thrombosis (10), which in medium sized vessels, such as the major coronary or cerebral vessels, becomes an obstructive atherothrombosis, causing ischaemia and eventual infarction of the tissue perfused by that vessel.

### Plaque Phenotypes

Atherosclerotic plaque is classified into stable and unstable phenotypes (3). The stable atherosclerotic plaque is characterised by a thick fibrous cap covering a small necrotic core, which can withstand haemodynamic changes and stresses and is therefore less susceptible to rupture (3, 12). In contrast, the unstable atherosclerotic plaque that is prone to rupture is associated with a thin fibrous cap covering a large necrotic core (10).

## IL-32

**Figure 1:**
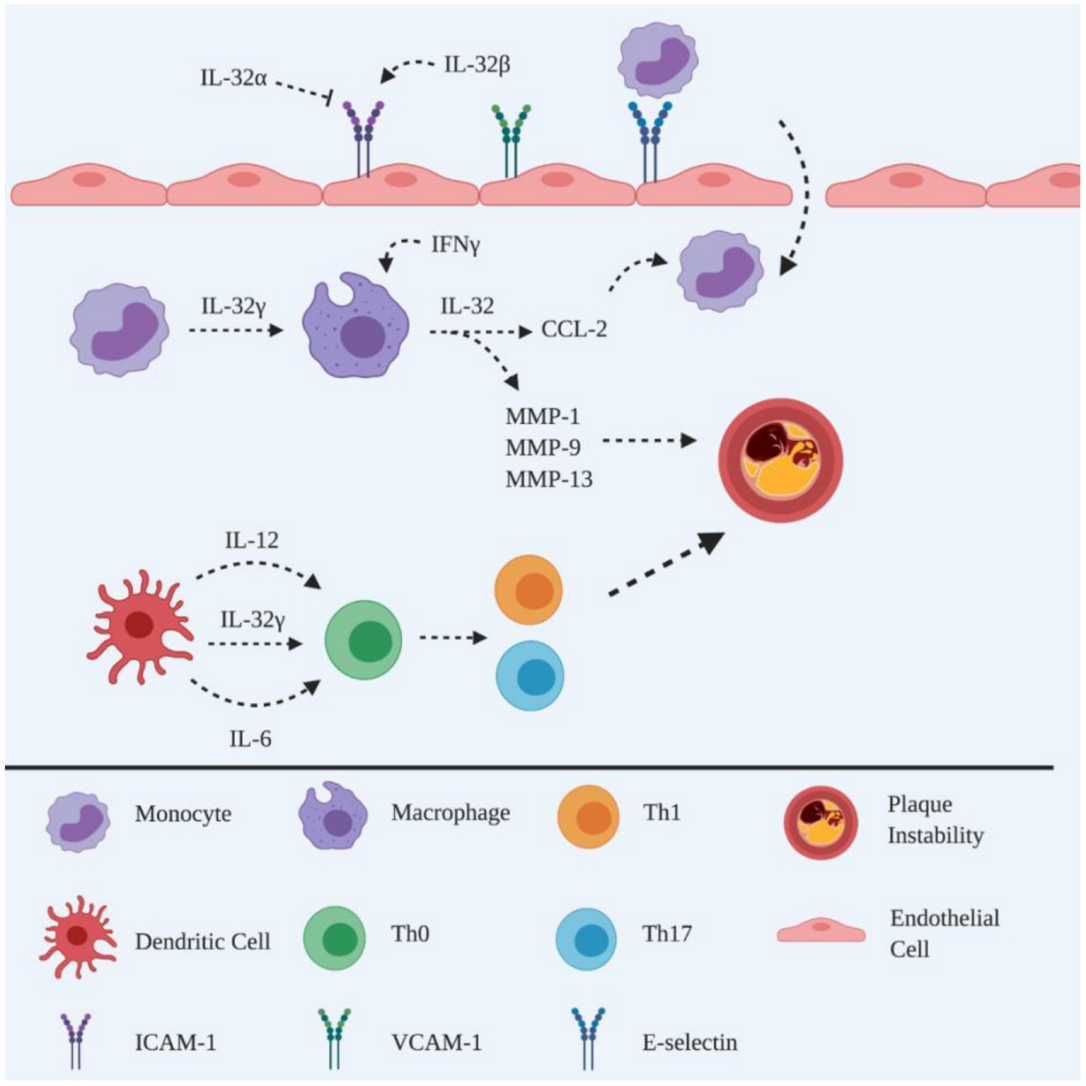
Schematic representation of the roles of IL-32 in atherogenesis. Adhesion molecules are promoted by IL-32β to facilitate monocyte recruitment, whereas recruitment can also be inhibited by IL-32α. The differentiation of monocytes into phagocytic macrophages is induced by IL-32γ, which in turn triggers the release of CCL-2 to recruit circulating monocytes. IL-32γ induces the maturation of DCs, releasing IL-12 and IL-6 to polarise naïve CD4^+^ T cells into Th1 and Th17 subsets. IL-32γ induces macrophages to produce MMPs leading to atherosclerotic plaque instability. Created with BioRender.com.

IL-32, formerly named natural killer cell transcript 4 (NK4), is constitutively produced by peripheral blood mononuclear (PBMC), epithelial and endothelial cells (13, 14). IL-32 consists of eight splice variants, however, only the IL-32α, IL-32β and IL-32γ isoforms have been extensively studied (15). An abundance of IL-32α is found in haematopoietic cells; whereas IL-32β and IL-32γ are the major isoform in endothelial cells and are the most active isoforms, respectively (13, 14, 16) (Figure 1).

Overexpression of IL-32 has been reported in rheumatoid arthritis (RA) (17) and Crohn's disease (18), as well as, in human symptomatic atherosclerotic plaques (19), compared to asymptomatic individuals (20). Interestingly, anti-inflammatory activity has been demonstrated in a murine model of asthma with allergic airways inflammation (21). Although the precise explanation for this apparent discrepancy in the activity of IL-32 remains unknown, it may be due to differences in inflammatory regulators between species and/or diseases.

### IL-32 and Atherogenesis

IL-32 has been detected in human endothelial cells of atherosclerotic plaques (22) and different isoforms have been demonstrated to exhibit distinct functional roles (23). IL-32α is associated with the suppression of ICAM-1 and VCAM-1 expression on endothelial cells, resulting in attenuation of atherosclerotic lesions, with decreased leukocyte infiltration being observed following overexpression of IL-32α in the IL-32α tg *Apoe*^−/−^ mouse model of atherosclerosis, suggesting that IL-32α is anti-inflammatory during atherogenesis (24). This is consistent with the finding that IL-32α enhances lipid accumulation and inhibits cholesterol efflux from ox-LDL-exposed THP-1 macrophages *via* the PPARγ-LXRα-ABCA1 pathway (25).

On the other hand, IL-32β promotes vascular inflammation, based on the observation of increased leukocyte adhesion on endothelial cells following overexpression of IL-32β in a transgenic mouse model of atherosclerosis (26), perhaps *via* upregulation of ICAM-1/VCAM-1 expression by IL-32β, as observed on human umbilical vein endothelial cells (HUVECs) following IL-32β stimulation (27). In addition, IL-32 regulates the function of endothelial cells within the aortic, coronary and pulmonary circulations, via IL-1β and other pro-inflammatory cytokines, particularly regulating I-CAM (27).

Thus, taken together, these data support the hypothesis that atherosclerotic development is accelerated by unbalanced expression of IL-32α and IL-32β facilitating vascular inflammation.

Furthermore, IL-32β and IL-32γ have been detected in macrophages of human atherosclerotic plaques, while IL-32γ is associated with greater MCP-1/CCL2 production from monocytic THP-1 cells, suggesting that IL-32γ amplifies local inflammation *via* recruitment of monocytes/macrophages (20). These data are consistent with the finding that IL-32γ enhances monocytes differentiation into macrophage-like cells (28), suggesting that IL-32γ is important for the regulation of the host response against antigens that the immune system detects within atherosclerotic plaques.

It is well known that macrophage heterogeneity is involved in atherogenesis, which consists of pro-inflammatory M1 and anti-inflammatory M2 macrophages (29). Interestingly, M2 macrophages shift towards a pro-atherogenic profile when in a pro-inflammatory micro-environment, as reported by the finding that M2 macrophages transform into foam cells *via* upregulation of scavenger receptor CD36 to internalise OxLDL at a higher capacity than M1 macrophages, following their exposure to OxLDL (30). In relation to the IL-32s, M2 rather than M1 macrophages demonstrate a significant upregulation of IL-32 expression in the presence of IFNγ, suggesting that IL-32 is an effector molecule mediating pro-atherogenic responses in the presence of pro-inflammatory stimuli (20). Since IL-32β is a less bioactive form, the upregulation of IL-32β in macrophages may be a form of reverse regulation that is generated by the alternative splicing of the IL-32γ transcript to reduce the overall pro-atherogenic effect (20).

The maturation of murine dendritic cells (DC) is promoted in the presence of rhIL-32γ (31). Specifically, rhIL-32γ increases the production of IL-12 and IL-6 in murine DCs, promoting the polarisation of CD4^+^ T cells into Th1 and Th17 subsets, accompanied by increased production of IFNγ and IL-17, respectively (31). This is an important mechanism in atherogenesis, in which IFNγ destabilises atherosclerotic plaques *via* the inhibition of VSMC proliferation leading to a thin fibrous cap (10). It is the degradation of the extracellular matrix, i.e., collagen, by matrix metalloproteinases (MMP) that causes thinning of the fibrous cap (3), which can be promoted by IL-32γ *via* increasing the secretion of MMP-1, MMP-9 and MMP-13 from macrophages (20). These data suggest that IL-32 contributes to plaque instability, which supports the finding of a strong correlation between IL-32 and symptomatic plaque phenotype in human atherosclerosis (19).

However, the more controversial role of IL-32, i.e., its anti-inflammatory role, has also been reported. It is well accepted that disruption of the removal of excessive cholesterol in the arterial wall is important in atherogenesis (2), which is regulated by the reverse cholesterol transport (RCT) mechanism *via* high density lipoproteins (HDL) transporting cholesterol to the liver for excretion (32). Increased HDL is associated with ameliorated human coronary atherosclerosis (32). Interestingly, increased HDL has been associated with an IL-32 promoter single nucleotide polymorphism (SNP) in rheumatoid arthritis patients (33), implying an anti-inflammatory role of IL-32 in CVD (33). This is supported by the findings that cholesterol is eliminated *via* ABCA-1, which can be induced by intracellular IL-32γ in hepatocytes (34). In the same study, both IL-32γ and ABCA-1 mRNA have been found in human carotid artery plaques (34). However, this relationship remains to be clarified, since this study did not show that IL-32γ and ABCA-1 can be colocalised *in vivo* in macrophages.

Taken together, the role of IL-32 during the development of atherosclerosis remains to be elucidated. However, we speculate that IL-32 acts differently in different stages of atherogenesis, perhaps depending on the different stimuli occurring within the plaque at various stages of development, based on the data described above. The precise underlying mechanism of IL-32 in atherogenesis, particularly in the presence of M1 vs M2 macrophages warrants further study.

## IL-34

**Figure 2:**
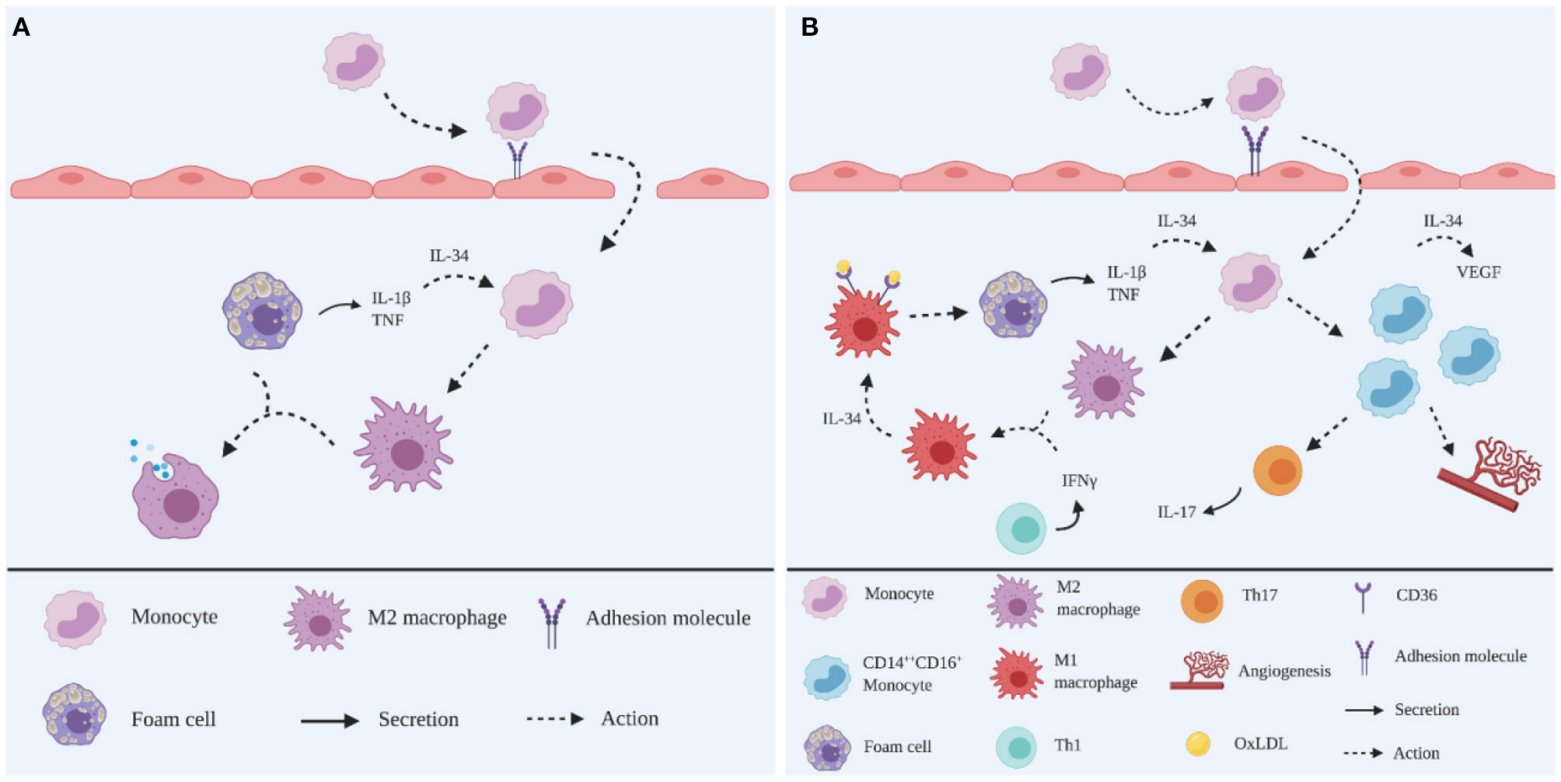
Schematic representation of the roles of IL-34 in atherogenesis. **(A)** In the early stage, TNF and IL-1β produced in the plaque microenvironment stimulate IL-34 production. Infiltrated monocytes are induced by IL-34 to differentiate into M2 macrophages to dampen the inflammatory responses by digesting OxLDL. **(B)** In the advanced stage, IFNγ produced from overwhelming inflammation skews M2 macrophages into an M1 phenotype. These M1 macrophages are induced by IL-34 to upregulate scavenger receptor CD36 to ingest OxLDL, leading to foam cell formation. IL-34 induces the expansion of CD14^bright^CD16^+^ monocytes subpopulations, increasing Th17 polarisation and angiogenesis, together with increased VEGF production. Created with BioRender.com.

IL-34 is a haematopoietic cytokine that shares similar functions with CSF-1/M-CSF, to maintain the viability of the myeloid cells lineage (35). Overexpression of IL-34 is associated with autoimmune diseases, such as RA (36), inflammatory bowel disease (IBD) (37) and Sjogren's syndrome (38). Upregulated IL-34 is also detected in human atherosclerotic plaques, particularly correlating with unstable plaques (19), suggesting that the pro-inflammatory activities of IL-34 in the advanced stages of plaque development may contribute to acute coronary syndrome and premature death (39). In addition, a substantial circulating IL-34 level has been detected in CAD patients and is associated with the severity of comorbid CAD in heart failure (40, 41) (Figure 2).

### Roles in Atherogenesis

IL-34 upregulates the scavenger receptor CD36 on murine bone-marrow derived macrophages to promote foam cell formation *via* the internalisation of OxLDL *in vitro* (42). In addition, IL-34 increases the mRNA expression of IL-1β, IL-6 and TNF in murine bone-marrow derived macrophages *in vitro* in the presence of OxLDL (42). These observations are consistent with the finding that IL-34 can elevate the production of chemokines and cytokines, including IL-6, in human PBMC (43). Moreover, IL-34 is upregulated in the presence of TNF and IL-1β (36, 38), suggesting IL-34 may act as a pro-atherogenic factor in both a paracrine and autocrine fashion to enhance foam cell formation in the plaque microenvironment.

Angiogenesis, which is known to promote plaque growth, is promoted in the presence of IL-34 *in vitro* (44, 45). Human PBMCs produce a significant level of VEGF in response to recombinant human (rh) IL-34 (45). Additionally, it is increasingly recognised that monocytes are classified into different subsets based on phenotypic characteristics and have distinct roles during the inflammatory response of atherosclerosis (46), including in relation to angiogenesis. Briefly, these subsets are: classical CD14^bright^CD16^−^, intermediate CD14^bright^CD16^+^ and non-classical CD14^dim^CD16^+^ monocytes, of which the intermediate CD14^bright^CD16^+^ monocytes are pro-atherogenic (46). It has also been shown that CD14^bright^CD16^+^ monocytes express vascular growth factor receptor-2 (VEGFR2) and respond to VEGF, suggesting a pro-angiogenic property (47). Since CD14^bright^CD16^+^ monocytes are abundantly detected in CAD patients (48), it is reasonable to speculate that IL-34 may promote angiogenesis *via* CD14^bright^CD16^+^ monocytes stimulation.

In addition, IL-34 induces Th17 polarisation, as evidenced by an increased Th17 cell population following the coculture of IL-34 treated macrophages and naïve CD4^+^ T cells (49). In the presence of IL-34, Th17 polarisation is promoted *via* upregulating IL-6 from human fibroblast-like synoviocytes (50). IL-23 has been shown to be produced by CD14^bright^CD16^+^ monocytes to induce Th17 polarisation *in vitro* (51). These observations correlate with the high expression of IL-34 in Sjogren's syndrome, in conjunction with an increased expression of IL-17 and IL-23 *in vivo*, suggesting that IL-34 may be linked to the IL-23/Th17 axis (38). Thus, it is reasonable to speculate that IL-34 induces Th17 polarisation during atherogenesis.

In contrast, IL-34 also exhibits an anti-inflammatory capacity. Human monocytes have been shown to differentiate into M2 macrophages in response to IL-34 *in vitro* (44, 52). Interestingly, M2 macrophages that are differentiated in the presence of IL-34, skew towards a pro-inflammatory M1 phenotype in response to IFNγ (52). This finding suggests that IL-34 plays an immunoregulatory role in the early stage of atherogenesis by inducing M2 macrophages to dampen the inflammatory responses and tissue remodelling. This is supported by the report from Boulakirba et al., showing IL-34 promotes M2 polarisation (53).

However, subsequently these M2 macrophages skew towards an M1 phenotype in response to increased IFNγ, which results from overwhelming inflammation in the plaque microenvironment.

Taken together, the role of IL-34 in atherogenesis remains ambiguous due to the complexity of the immune system. However, it is reasonable to suggest that the differential role of IL-34 in different stages of atherogenesis may depend on the specific anti-inflammatory or pro-inflammatory microenvironment in the early or advanced stages of atherogenesis.

## IL-37

IL-37 is an anti-inflammatory cytokine member of the IL-1 family (54, 55). IL-37 is constitutively expressed by immune cells including macrophages and DCs, as well as epithelial cells, and is upregulated in response to pro-inflammatory stimuli such as cytokines and TLR ligation (55). IL-37 functions through a heterodimeric receptor, which is composed of IL-18Rα and IL-1R8 (55). Elevated IL-37 expression is detected in autoimmune diseases such as RA (56) and IBD (57). Elevated IL-37 expression has also been observed in a murine model of atherosclerosis (58) as well as in plasma from acute coronary syndrome patients (59).

### IL-37 in Atherogenesis

#### IL-37 Host Immunity Mediated Atherogenesis

The activity of IL-37 was initially suggested to be pro-atherogenic because high levels of IL-37 are detected in foam cells within atherosclerotic plaques (59). However, interestingly, treatment with recombinant IL-37 has been shown to ameliorate the size of atherosclerotic plaque in diabetic *Apoe*^−/−^ mice, and is associated with increased anti-inflammatory IL-10, but not pro-inflammatory TNF or IL-18 (60). This striking finding is further supported by another study, showing that plaque size is reduced in IL-37 tg *Apoe*^−/−^ mice (61) and bone marrow transplanted *Ldlr*^−/−^ mice with increased endogenous IL-37 expression (62). Moreover, IL-37 reduces atherogenesis *via* decreasing circulating pro-inflammatory and increasing anti-inflammatory cytokines in IL-37 tg *Apoe*^−/−^ mice (63) and IL-37 treated *Apoe*^−/−^ mice (58).

Human coronary artery endothelial cells that have been transfected with IL-37 demonstrate downregulation of ICAM-1 in the presence of TLR2 ligand stimuli *in vitro* (64). IL-1β, which is known to upregulate adhesion molecules, is reduced in the presence of IL-37 in OxLDL-treated macrophages *in vitro* (62). These findings, in conjunction with evidence of reduced production of TNF and IL-1β, as well as reduced leukocytes infiltration, in the inflamed colon of IL-37 tg mice with colitis (65), suggest that IL-37 reduces leukocytes recruitment *via* downregulation of TNF and IL-1β during atherogenesis. Furthermore, IL-37-expressing mouse bone marrow-derived macrophages not only reduce uptake of OxLDL, but also decrease macrophage transmigration towards MCP-1 (62). These findings suggest that IL-37 plays an anti-atherogenic role *via* a negative regulatory mechanism to dampen the inflammation in atherosclerosis, perhaps by reducing foam cell formation, pro-inflammatory cytokines, as well as macrophage infiltration. The anti-inflammatory function of IL-37 during atherosclerosis is supported by data from others showing an inverse correlation between IL-37 and M1 macrophage polarisation in human calcified aortic valves (66), as well as in an animal atherosclerotic model (67), perhaps *via* suppressing M1 polarisation. However, while IL-37 reduces systemic inflammation, it does not influence atherosclerosis development in hyperlipidemic LDLr-deficient mice, which might be due to LDLr depletion (68). These mechanisms require future elucidation due to the potential for a major discrepancy between the human and murine context.

**Figure 3:**
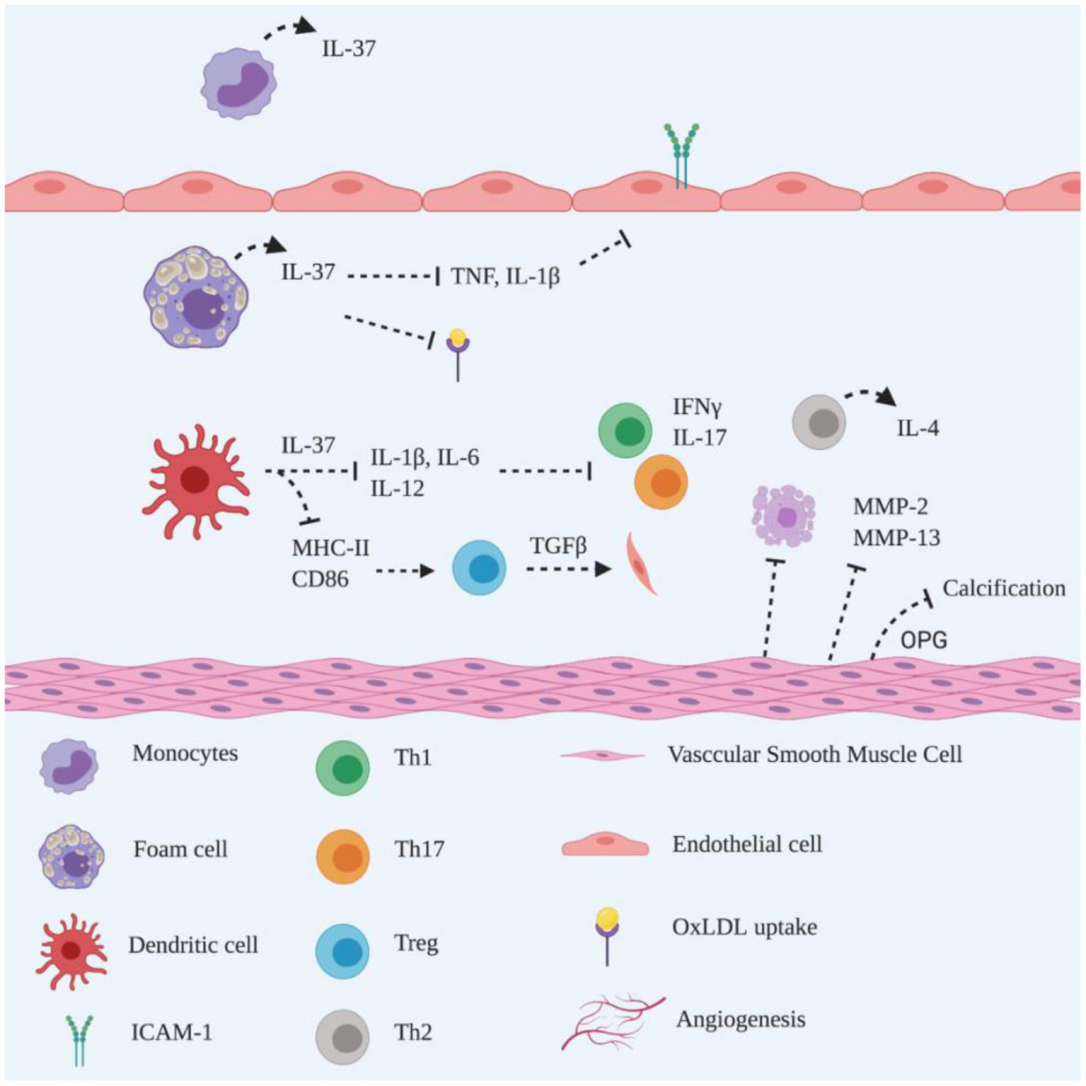
Schematic representation of the roles of IL-37 in atherogenesis. IL-37 is constitutively expressed by monocytes in the unstimulated state. In pathological conditions, IL-37 is upregulated by foam cells to suppress pro-inflammatory cytokines secretion and reduce OxLDL uptake and adhesion molecules expression on endothelial cells. IL-37 downregulates MHC-II and CD86 on dendritic cells to induce Treg activation, promoting collagen deposition via TGFβ production. Additionally, IL-37 reduces IL-1β, IL-6 and IL-12 production, to suppress Th1 and Th17 polarisation accompanied by reduced IFNγ and IL-17 secretion. It remains unclear whether the Th2 population is induced by dendritic cells or IL-37 producing T lymphocytes. IL-37 triggers VSMC to reduce MMP-2 and−13 production, attenuating collagen degradation and inhibiting apoptosis. IL-37 functions closely with VSMC-derived OPG, inhibiting vascular calcification. Created with BioRender.com.

IL-37 functions in a dual fashion in DCs to maintain an anti-inflammatory environment by implementing its anti-inflammatory actions intracellularly or by being released as a regulatory cytokine (69). Isolated bone marrow-derived DCs from IL-37 tg mice generate a tolerogenic phenotype in the presence of LPS by downregulating MHC-II and the costimulatory molecule CD40 (70). The findings which show the downregulation of MHC-II and CD86 in DCs from rhIL-37 treated *Apoe*^−/−^ mice (58) and IL-37 tg *Apoe*^−/−^ mice (63) suggest that atherogenesis is attenuated *via* reduced antigen presentation (Figure 3).

A reduction of Th1 cells is detected in rhIL-37 treated *Apoe*^−/−^ mice (58) and IL-37 tg *Apoe*^−/−^ mice (61), which is consistent with the observed reduction in Th1 cells in IL-37 treated splenic lymphocytes, which is accompanied by decreased IFNγ secretion (58, 61). However, there was no significant reduction of Th17 cells observed in the latter study (61), which suggests that IL-37 promotes Th polarisation during atherogenesis. T regulatory (Treg) cells play an athero-protective role in atherosclerosis *via* IL-10 inhibition of disease progression and TGFβ stimulation of collagen deposition to maintain plaque stability (10). The development of Treg cells is promoted in the presence of isolated bone marrow-derived DCs from IL-37 tg mice *in vitro* (70). This finding is supported by others, showing that Treg cells are increased in rhIL-37 treated *Apoe*^−/−^ mice *in vivo* and increased production of TGFβ and IL-10 is induced during the coculture of CD4^+^ T cells with OxLDL plus IL-37-treated bone marrow-derived DCs (58). Interestingly, Th2 cells, but not Treg cells, together with IL-4, are abundant in IL-37 tg *Apoe*^−/−^ mice (61), suggesting that different signalling mechanisms may be exerted by exogenous and/or endogenous IL-37. CD4^+^ T cells have been shown to be the major source of IL-37 in human atherosclerotic plaques (58, 61). Since Th1 cells shift towards Th2 cells in the presence of IL-37 *in vitro* (61), the hypothesis emerges that Th2 polarisation may be spontaneously induced by CD4^+^ T cell-derived IL-37 in the plaque microenvironment. These data are in line with others who have shown that IL-37 contributes to the anti-inflammatory response in the development of atherosclerosis, perhaps *via* enhancing Treg cells (71). Interestingly, elevated circulating and local IL-37 in atherosclerotic rabbits is suppressed by atorvastatin (72), suggesting that atorvastatin dampens systemic and local inflammation, resulting in a reduction of IL-37.

#### IL-37 and Plaque Stability

It is recognised that plaque vulnerability is also promoted by VSMC apoptosis (73). IL-37 inhibits VSMC apoptosis, as evidenced by the reduced apoptotic VSMC area in atherosclerotic plaques of IL-37 tg *Apoe*^−/−^ mice (61). Such findings are supported by attenuated atherosclerotic plaque in rhIL-37 treated *Apoe*^−/−^ mice, showing a larger VSMC- and collagen-positive staining area than a mock treated group (58). An increased amount of collagen content, with reduced mRNA expression of MMP-2/-13 within atherosclerotic plaque has been observed in IL-37 tg *Apoe*^−/−^ mice, compared to *Apoe*^−/−^ mice only (61), suggesting that IL-37 plays an important role in maintaining plaque stability. VSMC proliferation is reparative and advantageous for atherogenesis in both early and advanced stages, to maintain plaque stability (74). As IL-37 is expressed by VSMC to maintain plaque stability in human atherosclerotic plaques (58, 61), it is reasonable to speculate that IL-37 also induces VSMC proliferation *via* an autocrine mechanism.

Vascular calcification is also one of the key features of atherosclerosis and serves as an independent predictor for acute coronary events (75). Spotty microcalcifications that are dispersed within the necrotic core and fibrous cap drive plaque instability (75). It is well recognised that calcification is driven by VSMC plasticity *via* trans-differentiation into osteoblast, chondrocyte and macrophage-like phenotypes in response to pro-inflammatory cytokines in atherosclerotic plaques, which release pro-calcific factors accompanied by a loss of calcification inhibitors (76). Reduced calcification in the aortic root has been observed in rhIL-37 treated *Apoe*^−/−^ mice (60), which is consistent with findings in humans, where IL-37 is highly detected in calcified human aortic valve interstitial cells *in vivo*, as well as reduced calcification in calcified human aortic valve interstitial cells in the presence of rIL-37 *in vitro* (77). Osteoprotegrin (OPG), which is a calcification inhibitor, is highly detected in VSMCs of atherosclerotic plaques in rhIL-37 treated *Apoe*^−/−^ mice (60). However, in the presence of anti-OPG antibody, increased calcified areas are observed, implicating a close relationship between IL-37 and OPG for calcification regulation (60). These finding are indirectly supported by the observation that IL-37 is abundantly detected in human calcified coronary arteries, particularly in VSMCs, compared to normal arteries, suggesting that the purpose of upregulation of IL-37 is to alleviate arterial calcification (78). In addition, a positive correlation between plasma IL-37 and OPG has been detected in patients with severe coronary artery calcification, suggesting that IL-37 is a potential biomarker of arterial calcification (79).

Since an effective treatment to mitigate vascular calcification remains undetermined (75, 76), investigation of the underlying mechanisms of IL-37 in VSMC may provide future therapeutic opportunities.

In addition elevated plasma IL-37 has been detected in acute ischemic stroke patients, and IL-37 is an independent association with poorer prognoses (80), which is consistent with others, showing elevated circulating IL-37 is associated with a poor outcome in ST-segment elevation acute myocardial infarction in acute coronary syndrome patients (81, 82), although this finding remains controversial (83).

Taken together, IL-37 plays an anti-atherogenic role in atherogenesis. Although the exact mechanism is not well understood, data support speculation that elevation of IL-37 expression is a compensatory mechanism to suppress plaque inflammation, however, inflammatory cells may fail to respond effectively to IL-37 due to exhaustion or the complex nature of the plaque microenvironment, resulting in a continuous release of ineffective IL-37. In relation to COVID-19, IL-37 has been suggested to be a potential treatment based on its anti-inflammatory profile to inhibit IL-1β, IL-6 and TNF, which are the main players of the cytokine storm (84).

## Clinical Implications of IL-32, IL-34 and IL37 in Atherosclerosis

The role of IL-32 during the development of atherosclerosis has been illustrated, showing that IL-32 promotes angiogenesis on endothelial cells, suggesting IL-32 boosts the development of atherosclerosis (85). This is in line with others, showing that the protective role of IL-32 during the development of atherosclerosis is related to a single promoter single-nucleotide polymorphism (SNP) in IL-32, contributing to modified lipid profiles, especially in rheumatoid arthritis patients (33). Furthermore, the benefit of the SNP in IL-32 is related to reduce pro-inflammatory cytokines and increases HDLc concentration (15), further supporting the role of IL-32 during atherogenesis. This may also in line with the findings following influenza viral challenge, showing that increased IL-32 is beneficial against the viral infection (86).

The role of IL-34 during the development of atherosclerosis has been demonstrated, since there is an association between the level of IL-34 and severity of coronary artery disease in patients with heart failure, and IL-34 is an independent risk factor for CAD among heart failure patients, regardless of the systolic function (41). In addition, there is evidence from others, showing that IL-34 is significantly induced in influenza infected patients in an autocrine and paracrine fashion (87), supporting a role for IL-34 in the course of SARS-COV-2 viral infection. Furthermore, the possible mechanisms utilised by IL-34 in atherogenesis have been demonstrated via a linkage among IL-34, obesity, chronic inflammation, and insulin resistance, suggesting that IL-34 enhances atheroma *via* insulin resistance in obese patients (88).

Finally, increased circulating IL-37 levels have been correlated with high coronary calcium score levels, suggesting that IL-37 may contribute to the activation of inflammation. Furthermore, IL-37 has been proposed as a predictor of severe coronary artery disease (79). In addition, the importance of elevated serum and urine IL-37 has been demonstrated in post-ischemic stroke patients (89). However, it is unclear whether the increased IL-37 results from or results in such clinical manifestations. The possible mechanism of the anti-inflammatory role of IL-37 may be by antagonising inflammatory responses while retaining type I interferon, subsequently maintaining the functionalities of vital organs (90). The role of IL-37 in COVID-19 is supported by the findings in influenza viral infection, showing that IL-37 ameliorates influenza pneumonia *in vivo* (91). However, we have reviewed the mechanisms of action of IL-32, -34 and -37 in atherosclerosis, allowing us to speculate on the possible pathogenesis of SARS-CoV-2 involvement in CVD.

## Speculative Role of IL-32, IL-34 and IL-37 in Atherosclerosis and Covid-19

COVID-19 is caused by severe acute respiratory syndrome coronavirus 2 (SARS-CoV-2) (92), which is similar to severe acute respiratory syndrome coronavirus (SARS-CoV) (92) and Middle East respiratory syndrome coronavirus (MERS-CoV) (93). SARS-CoV-2 infects host cells by binding to the cell surface receptor angiotensin converting enzyme 2 (ACE2) receptor *via* the viral spike (S) protein (92). The original COVID-19 was first reported in Wuhan (94), then other regions of China (95, 95) and the became a pandemic (96).

Based on the current information available, during the course of COVID-19, particularly in moderate to severe COVID-19 patients, there is likely to be a contribution of COVID-19 in atherosclerosis, perhaps due to the cytokine storm causing vascular dysfunction via the ACE2 pathway, which likely further enhances local inflammation (97) and subsequently results in further activation of endothelial cells in large vessels (98), in addition to the microvascular system. Such insults from the cytokine storm also contribute to hyper-coagulation (99), but this will not be discussed further in the current review.

The role of IL-32 may be induced in local macro-vessels and micro-vessels, which may be due to SARS-COV-2 viral challenge via the ACE2-spike protein pathway. IL-32 may contribute to quench both systemic and local inflammation, which may be effective in moderate COVID-19 patients, but likely fails in severe patients. Subsequently, major organ failure would be induced due to infarction, e.g., heart, lung and kidney (100), particularly in the more susceptible COVID-19 patients. This speculation is supported by others, who have shown that steroids may help to reduce clinical symptoms and shorten the course of COVID-19 (101).

In contrast, IL-34 may contribute to atherosclerosis, but its role in COVID-19 remains unclear. We believe that IL-34 would be secreted by infiltrating inflammatory leucocytes, particularly macrophages and lymphocytes following the cytokine storm in COVID-19 patients (102). More obvious vascular manifestations would then result.

It has been reported that circulating IL-37 is elevated in COVID-19 infected patients. Interestingly, the patients with higher IL-37 had a shorter hospitalisation period than the lower group, suggesting that IL-37 may provide protection during the course of COVID-19 infection (90).

However, there is not yet any solid evidence to clearly state the direct involvement among IL-32, 34 and 37 in the atherogenesis in COVID-19 patients.

In addition there is a strong association between cardiovascular disease (CVD) and the susceptibility to, and the outcomes of, COVID-19 (103), including coronary artery disease (CAD), particularly among those patients with co-existing diabetes mellitus (104). Patients with pre-existing CVD, including hypertension, coronary artery disease (CAD) and diabetes mellitus are more susceptible to SARS-CoV-2 infection and are more likely to develop exaggerated cardiovascular sequelae (105), hence there is a higher prevalence of severe disease in the elderly population (106). A major contributing factor to the higher susceptibility among patients with pre-existing CVD is the higher levels of cell surface expression of ACE2, which makes the patients more vulnerable to SARS-CoV-2 viral infection (106, 107). Additionally, a small proportion of young adults without pre-existing CVD also develop cardiovascular complications following SARS-CoV-2 infection (108), which may be related to their exaggerated host immunity (cytokine storm) (109). One of the key contributing factors for the higher mortality and morbidity in COVID-19 patients is excess local production of pro-inflammatory cytokines, such as IL-1β, IL-6, IL-8 and TNF in key organs (heart, lungs and liver) (110–112), which is termed a cytokine storm (113). Consequently, substantial damage occurs in the heart, lungs, liver and kidneys, which contributes to the disease severity in COVID-19 patients (110). Although the underlying mechanism of SARS-CoV-2 viral attack is not well understood, these findings above suggest that a relationship exists between COVID-19 and CVD outcomes that is both bidirectional and multifactorial (106, 114). Thus, it is reasonable to speculate that many COVID-19-related heart problems are due to a cytokine storm, either in the heart or major arteries (115).

Interestingly, there is some limited data emerging in the literature supporting the view that COVID-19 may increase the rate of acute plaque rupture (116, 117). Respiratory infections such as influenza are known to be capable of triggering acute coronary syndrome (118), so it is likely that COVID-19 will act in a similar manner. A recent case report of an ACS event during COVID-19 infection supports this likelihood (116). Similarly, the likely mechanisms underpinning increased plaque instability during COVID-19 infection have been explored (107, 117).

## Conclusion

We conclude that IL-32 provides athero-protection *via* differential regulation of polarisation of macrophages in different stages of atherogenesis, perhaps depending on the different stimuli occurring within the plaque at various stages of development. Subsequently IL-32 down-regulates the activities of CCL-2 and MMPs, and finally ABCA1 pathway

IL-34 is pro-atherogenic and its role is stage dependent. In the early stage, recruited monocytes are induced by IL-34 to differentiate into M2 macrophages to dampen the inflammation in the presence of stimuli, e.g., OxLDL, in an autocrine and paracrine fashion. In the advanced stage, particularly in some SNP populations, macrophages are skewed towards the M1 phenotype, especially in the presence of a large amount of IFNγ. IL-34 induced M1 macrophages upregulate scavenger receptor CD36 to ingest OxLDL, leading to foam cell formation. Subsequently, IL-34 induces the expansion of CD14^bright^CD16^+^ monocytes subpopulations, further boosting the pro-inflammatory responses, including increasing Th17.

IL-37 is also athero-protective. Constitutively expressed IL-37 can be upregulated by foam cells to dampen proinflammatory cytokines secretion, reduce OxLDL uptake and adhesion molecules expression on endothelial cells, as well as downregulate MHC-II and CD86 on dendritic cells to induce Treg activation *via* TGFβ production. In addition, IL-37 reduces IL-1β, IL-6 and IL-12 to suppress Th1/Th17 polarisation, and subsequently down-regulates IFNγ and IL-17 secretion. IL-37 also reduces MMPs on VSMC and attenuates collagen degradation and inhibits apoptosis. Finally, IL-37 inhibits vascular calcification via VSMC-derived OPG.

Finally IL-32 and IL-37 may be protective while IL-34 may contribute to the development of atherosclerosis. In addition, we speculate that the role of IL-32 and 37 may also be beneficial, but IL-34 may be harmful, during the course of COVID-19. Such information highlights gaps in our current understanding for future studies to investigate. Our figures offer a very dynamic summary of these cytokines during the development of atherosclerosis. We believe that our review provides more in-depth information for both basic scientists and clinicians.

## Author Contributions

CL: conceptualised, drafted, and wrote the manuscript. RP and JFa: conceptualised. JFe: revised the manuscript. BH: revised and edited the manuscript. SB: conceptualised, drafted, and edited the manuscript. All authors contributed to the article and approved the submitted version.

## Conflict of Interest

The authors declare that the research was conducted in the absence of any commercial or financial relationships that could be construed as a potential conflict of interest.

## Publisher's Note

All claims expressed in this article are solely those of the authors and do not necessarily represent those of their affiliated organizations, or those of the publisher, the editors and the reviewers. Any product that may be evaluated in this article, or claim that may be made by its manufacturer, is not guaranteed or endorsed by the publisher.
